# Potential biomarkers for inflammatory response in acute lung injury

**DOI:** 10.1515/med-2022-0491

**Published:** 2022-06-08

**Authors:** Lanzhi Zheng, Zhuoyi Zhang, Kang Song, Xiaoyang Xu, Yixin Tong, Jinling Wei, Lu Jiang

**Affiliations:** Emergency Department, The First Affiliated Hospital of Zhejiang Chinese Medical University, Hangzhou City, 310006 Zhejiang Province, China; Emergency Department, The First Affiliated Hospital of Zhejiang Chinese Medical University, Youdian Road 54#, Shangcheng District, Hangzhou City, 310006 Zhejiang Province, China

**Keywords:** molecular biology, acute lung injury, inflammatory response, microRNAs

## Abstract

Acute lung injury (ALI) is a severe respiratory disorder occurring in critical care medicine, with high rates of mortality and morbidity. This study aims to screen the potential biomarkers for ALI.

Microarray data of lung tissues from lung-specific geranylgeranyl pyrophosphate synthase large subunit 1 knockout and wild-type mice treated with lipopolysaccharide were downloaded. Differentially expressed genes (DEGs) between ALI and wild-type mice were screened. Functional analysis and the protein–protein interaction (PPI) modules were analyzed. Finally, a miRNA-transcription factor (TF)-target regulation network was constructed.

Totally, 421 DEGs between ALI and wild-type mice were identified. The upregulated DEGs were mainly enriched in the peroxisome proliferator-activated receptor signaling pathway, and fatty acid metabolic process, while downregulated DEGs were related to cytokine–cytokine receptor interaction and regulation of cytokine production. *Cxcl5*, *Cxcl9*, *Ccr5*, and *Cxcr4* were key nodes in the PPI network. In addition, three miRNAs (miR505, miR23A, and miR23B) and three TFs (PU1, CEBPA, and CEBPB) were key molecules in the miRNA-TF-target network. Nine genes including *ADRA2A*, *P2RY12*, *ADORA1*, *CXCR1*, and *CXCR4* were predicted as potential druggable genes.

As a conclusion, *ADRA2A*, *P2RY12*, *ADORA1*, *CXCL5*, *CXCL9*, *CXCR1*, and *CXCR4* might be novel markers and potential druggable genes in ALI by regulating inflammatory response.

## 1 Introduction

As a clinically severe respiratory disorder, acute lung injury (ALI) usually occurs in critical care medicine caused by various direct or indirect injury factors, and further progresses to acute respiratory distress syndrome (ARDS), with high rates of mortality and morbidity [1,2]. ALI is the most severe form of the viral infection sustained by acute respiratory syndrome coronavirus-2 (SARS-CoV-2) [3,4]. ALI clinically manifests as progressive hypoxemia and respiratory distress due to alveolar barrier dysfunction and formation of alveolar edema [1,4]. Despite the intensive advances in modern treatment technology and the increased understanding of pathogenesis, significant mortality caused by ALI remains a serious issue [5,6]. Therefore, it is important to further reveal novel biomarkers and therapeutic methods, as well as the underlying mechanisms of ALI.

With the development of high-throughput sequencing and the continuous updating of bioinformatics technology, a large number of noncoding RNAs such as microRNAs (miRNAs) have been found to be predominately correlated with a wide variety of diseases [7]. miRNAs are arbitrarily defined as endogenous small-molecule RNAs with the length of 20–25 nucleotides, and exert biological functions by impeding translation of target mRNAs [8]. It is now well appreciated that miRNAs participate in disease progression by modulating various key cellular biological processes, including cell proliferation, differentiation, apoptosis, migration, and invasion [8]. To date, miRNAs have revealed abnormal expression in various diseases, and some of which serve as biomarkers for the targeted treatment of ALI [9]. Notably, it has been reported that statins are effective drugs for the treatment of ALI and coronavirus disease 2019 (COVID-19) by inhibiting the mevalonate pathway, which is a druggable target for COVID-19 [10,11]. As downstream of the mevalonate pathway, geranylgeranyl pyrophosphate synthase large subunit 1 (GGPPS1) is considered a target to treat lung fibrosis [12]. Recent studies have also demonstrated that lung-specific GGPPS1 knockout can attenuate lung inflammation and injury in lung injury mice [13,14]. However, few studies have investigated the underlying mechanism of ALI with GGPPS1-knockout.

Herein, we downloaded the microarray data of ALI, and the differentially expressed genes (DEGs) between ALI lung specimens from lung-specific GGPPS1-knockout and wild-type mice treated with lipopolysaccharide (LPS) were screened. Functional analysis was further explored to investigate the functional biological annotations associated with DEGs related to ALI. Meanwhile, to further explore the functional network of DEGs, the networks of protein–protein interaction (PPI), miRNA-transcription factor (TF)-target regulation, and the drug–gene interaction were predicted.

## 2 Materials and methods

### 2.1 Data acquisition

The gene expression profile dataset GSE89311 was downloaded from the National Center of Biotechnology Information Gene Expression Omnibus database (http://www.ncbi.nlm.nih.gov/geo/) on September 20, 2020. The dataset was based on the platform of GPL10787 Agilent-028005 SurePrint G3 Mouse GE 8x60K Microarray (Probe Name version). The GSE89311 dataset included eight lung tissues isolated from GGPPS1-knockout C57BL/6 mice (*n* = 4) treated with LPS for 12 h and wild-type C57BL/6 mice (*n* = 4) treated with LPS for 12 h.

### 2.2 Data preprocessing and DEG screening

The preprocessing of data was performed by the Limma package [15] of R software, which mainly consisted of background correction by MAS method, normalization by quantile methods, and expression calculation. When the probe was not mapped to any gene symbol, this probe would be removed from our analysis. When, however, one gene was mapped by multiple probes, the mean value of the probes was considered the expression value of this gene. Subsequently, the classical Bayes method provided by Limma package was employed to screen DEGs between GGPPS1-knockout and wild-type mice. Notably, the DEGs in this study were defined according to the cutoffs of *P* value < 0.05 and |log_2_(fold change)| > 1.0. Bidirectional clustering heatmap and volcano plots were constructed based on DEGs.

### 2.3 Gene Ontology (GO) and Kyoto encyclopedia of genes and genomes (KEGG) pathway enrichment analyses

To assess functions and significantly enriched pathways of DEGs, GO functional annotation correlated with GO biological process analysis and KEGG pathway enrichment analysis were conducted with clusterProfiler [16]. *p* value <0.05 and count ≥5 were regarded as thresholds for enrichment analyses.

### 2.4 PPI network construction and module analysis

The Search Tool for the Retrieval of Interacting Genes/Proteins (STRING) database (Version: 10.0, http://www.string-db.org/) [17] provides PPI prediction function and is available online. Therefore, we conducted the PPI analysis of DEGs on the basis of this database using the PPI score of 0.7 (high confidence) and expected to identify crucial protein pairs. Afterward, the PPI network was constructed and visualized using the Cytoscape software (version: 3.2.0, http://www.cytoscape.org/). Moreover, the CytoNCA plugin (version 2.1.6, http://apps.cytoscape.org/apps/cytonca) [18] was used to analyze the network topology properties of the nodes with the parameters of without weight. The important nodes involved in PPI, namely the hub proteins, were obtained by ranking the network topological properties of each node. Furthermore, the molecular complex detection (MCODE, version 1.4.2, http://apps.cytoscape.org/apps/MCODE) [19] plugin of Cytoscape was used to analyze modules with similar functions in the original PPI network. GO biological process and KEGG pathway analyses of modules were further performed to evaluate the functions of sub-modules.

### 2.5 Prediction of miRNA-TF-target regulation

Overrepresentation Enrichment Analysis (ORA) enrichment method through WebGestalt (http://www.webgestalt.org/) [20] was used to perform TF-target and miRNA-target enrichment prediction from all module genes. Next, the miRNA-TF-target relationship pairs were obtained based on the threshold of *P* value <0.05, and the network software was constructed using Cytoscape.

### 2.6 Prediction of drug–gene interaction

The Drug–Gene Interaction Database (DGIdb) is used to mine existing resources and generate assumptions about how genes are therapeutically targeted or prioritized for drug development. Based on module genes, drug–gene interaction was predicted by DGIdb2.0 (http://www.dgidb.org/) [21]. We only screened for FDA-approved drugs to predict all drug–gene relationship pairs. Meanwhile, the drug–gene interaction network was carried out and visualized by Cytoscape software (version: 3.2.0, http://www.cytoscape.org/). Because the database defaults to human genes, the module genes were transformed into mouse–human homologous genes, and the transformed genes were used to predict drug–gene interaction.

### 2.7 Selection of genes associated with lung cancer prognosis

The potential association of key hub genes in ALI with the prognosis of lung cancers was analyzed to investigate the crucial roles of these genes in lung diseases. The lung adenocarcinoma (LUAD) and lung squamous cell carcinoma (LUSC) data in the GEPIA online tool (http://gepia.cancer-pku.cn/) were used for the survival analysis. The association of gene expression with the overall survival of LUAD and LUSC patients was extracted. Significant correlation was regarded as logrank *P* value <0.05.

## 3 Results

### 3.1 Identification of DEGs

**Figure 1:**
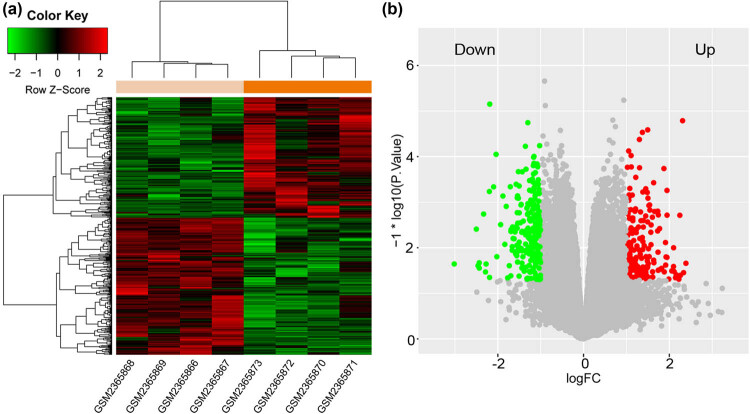
DEGs between ALI tissue specimens from lung-specific GGPPS1-knockout mice and wild-type mice. FC, fold change. (a) Bidirectional clustering heat map. (b) Volcano distribution map.

Totally, 421 DEGs (including 224 downregulated and 197 upregulated) were identified in lung samples isolated from GGPPS1-knockout mice treated with LPS for 12 h compared with wild-type mice treated with LPS for 12 h (Figure 1a and b).

### 3.2 Functional enrichment analysis of DEGs

**Figure 2:**
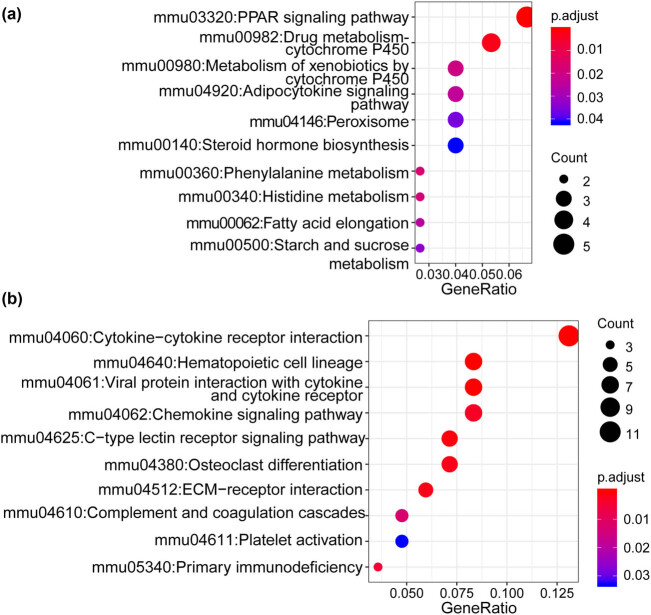
The KEGG pathway enrichment analysis of DEGs (a) and (b). The top ten KEGG pathways of upregulated and downregulated DEGs, respectively.

It was observed that upregulated DEGs were associated with 14 KEGG pathways, including “mmu04060:Cytokine–cytokine receptor interaction,” “mmu03320:PPAR signaling pathway,” “mmu04920:Adipocytokine signaling pathway,” “mmu00140: Steroid hormone biosynthesis,” “mmu00062:Fatty acid elongation,” and “mmu00500:Starch and sucrose metabolism” (Figure 2a). Downregulated DEGs were enriched in ten KEGG pathways, including “mmu04380:Osteoclast differentiation,” “mmu04062:Chemokine signaling pathway,” “mmu04060:Cytokine−cytokine receptor interaction,” “mmu04512:ECM–receptor interaction,” and “mmu04611:Platelet activation” (Figure 2b). These results suggested the differentially biological functions by the upregulated and downregulated DEGs.

### 3.3 PPI network analysis

**Figure 3:**
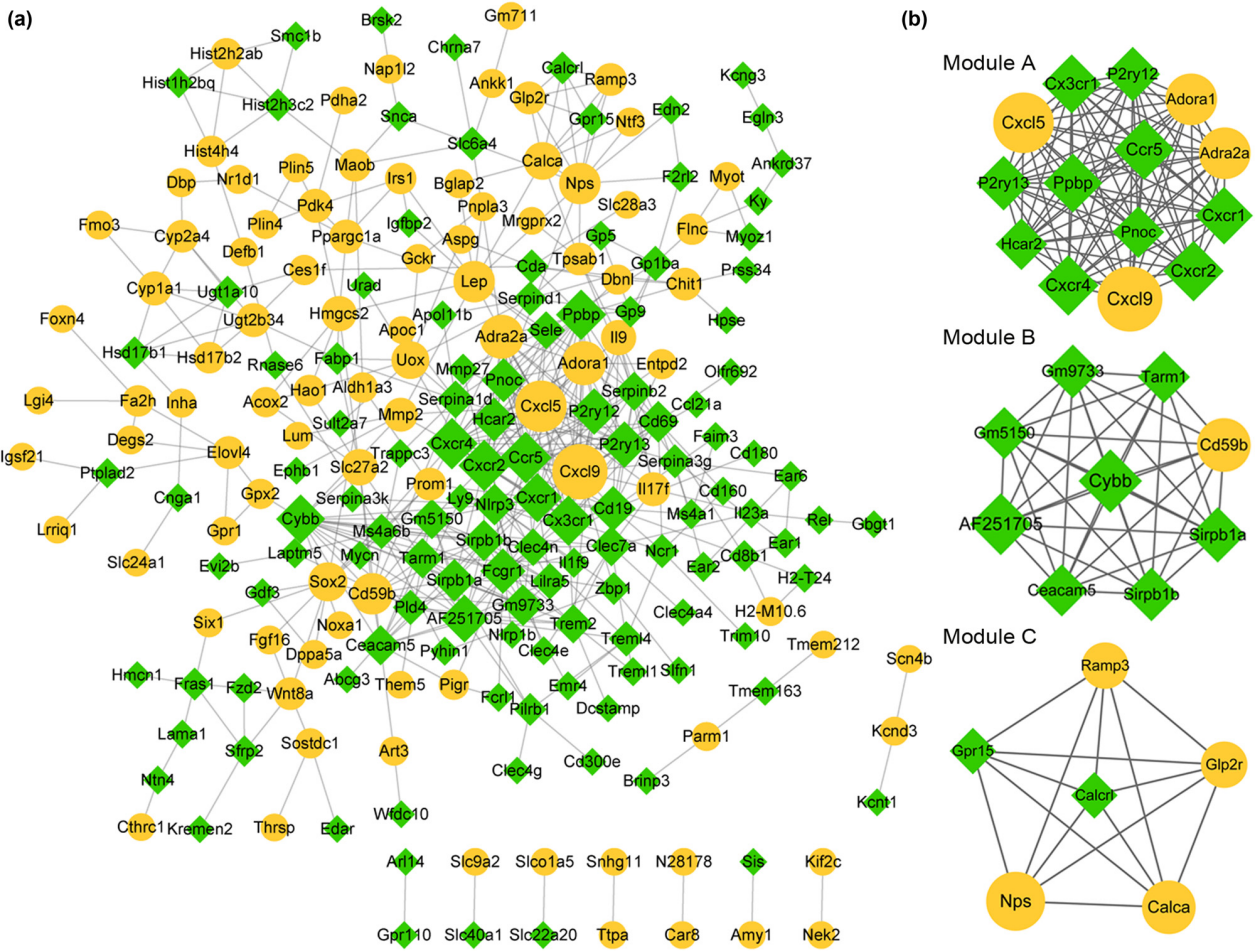
The PPI network of different expressed genes (DEGs). (a) and (b) The PPI network and three sub-modules of DEGs, respectively. The yellow circular node represents upregulated genes; the green prismatic node represents downregulated genes.

**Figure 4:**
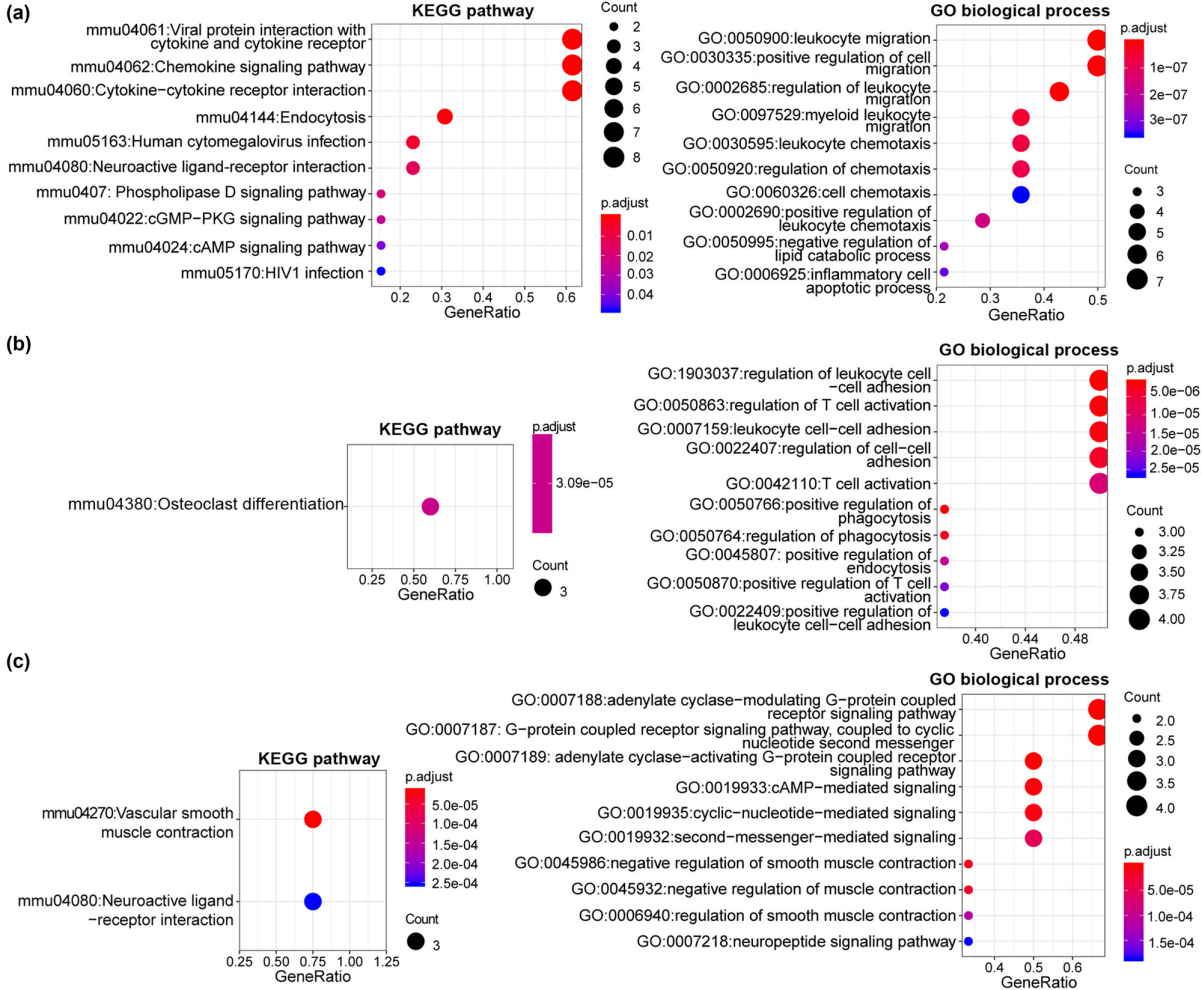
The enrichment of different expressed genes (DEGs). (a) The top ten KEGG pathways and GO biological process terms of DEGs in module-A. (b) One KEGG pathway and top ten GO biological process terms of DEGs in module-B. (c) Two KEGG pathways and top ten GO biological process terms of DEGs in module-C.

PPI network was undertaken using the STRING database to explore the PPI relationships of the overlapping DEGs. Overall, 216 nodes and 504 protein pairs were achieved (Figure 3a). Furthermore, the module analysis of PPI network indicated three sub-modules, and *Cxcl9*, *Ccr5*, *Cxcr4*, *Cxcl5*, *Sirpb1a*, *Sirpb1b*, *Nps*, *Calca*, and *Ramp3* were key nodes in these modules (Figure 3b). Specifically, module-A (score = 14) contained 14 nodes and 91 protein pairs. Genes in module-A were mainly associated with viral protein interaction with cytokine and cytokine receptor, chemokine signaling pathway, leukocyte migration, and regulation of cell migration based on the analysis of KEGG pathways and GO biological processes (Figure 4a). Also, one upregulated DEG and eight downregulated DEGs were included in module-B (score = 9), and they were primarily implicated with one KEGG pathway of “mmu04380:Osteoclast differentiation” and the GO biological processes related to regulation of cell–cell adhesion, endocytosis, T cell activation, and leukocytes (Figure 4b). Genes in module-C (score = 6; four upregulated genes and two downregulated) were prominently related to two KEGG pathways of “mmu04270:Vascular smooth muscle contraction” and “mmu04080:Neuroactive ligand–receptor interaction” and GO biological processes related to regulation of smooth muscle contraction and cAMP-mediated signaling (Figure 4c).

### 3.4 MiRNA-TF-target network

**Figure 5:**
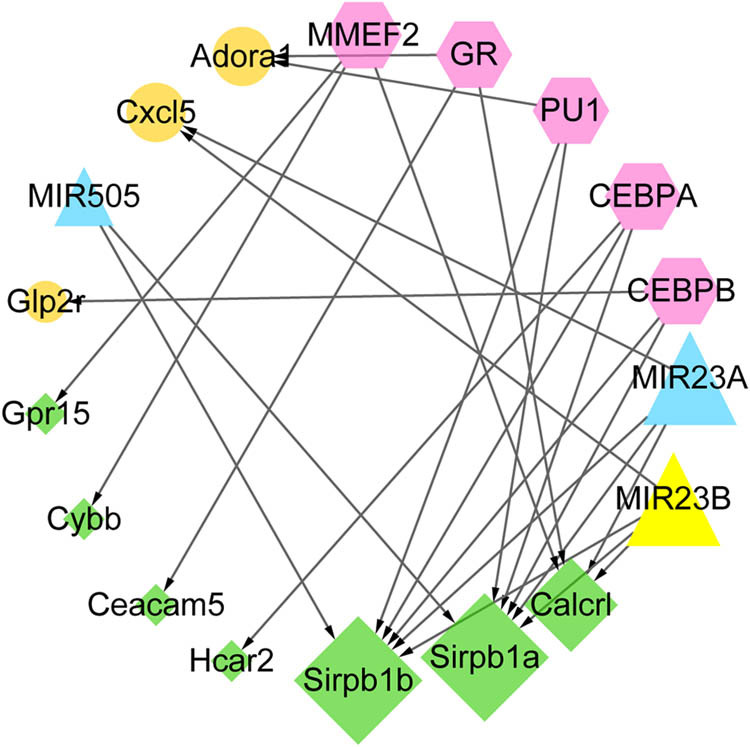
Analysis of TF-miRNA-target gene regulatory network. The yellow circle represents the upregulated gene, the green prismatic represents downregulated gene. The red hexagon represents microRNA (miRNA), the blue triangle represents TF, and the arrow connecting line indicates the direction of regulation.

Based on WebGestalt prediction, the miRNA-TF-target network was constructed. Three miRNAs (miR505, miR23A, and miR23B), five TFs (MMEF2, glucocorticoid receptor, PU1, CCATT/enhancer binding protein alpha [CEBPA], and CEBPB), three upregulated DEGs (*Adora1*, *Cxcl5*, and *Glp2r*), and seven downregulated DEGs (*Gpr15*, *Cybb*, *Ceacam5*, *Hcar2*, *Sirpb1b*, *Sirpb1a*, and *Calcr1*) were included, integrating with 25 regulatory relationship pairs (Figure 5).

### 3.5 Drug–gene interaction network

**Figure 6:**
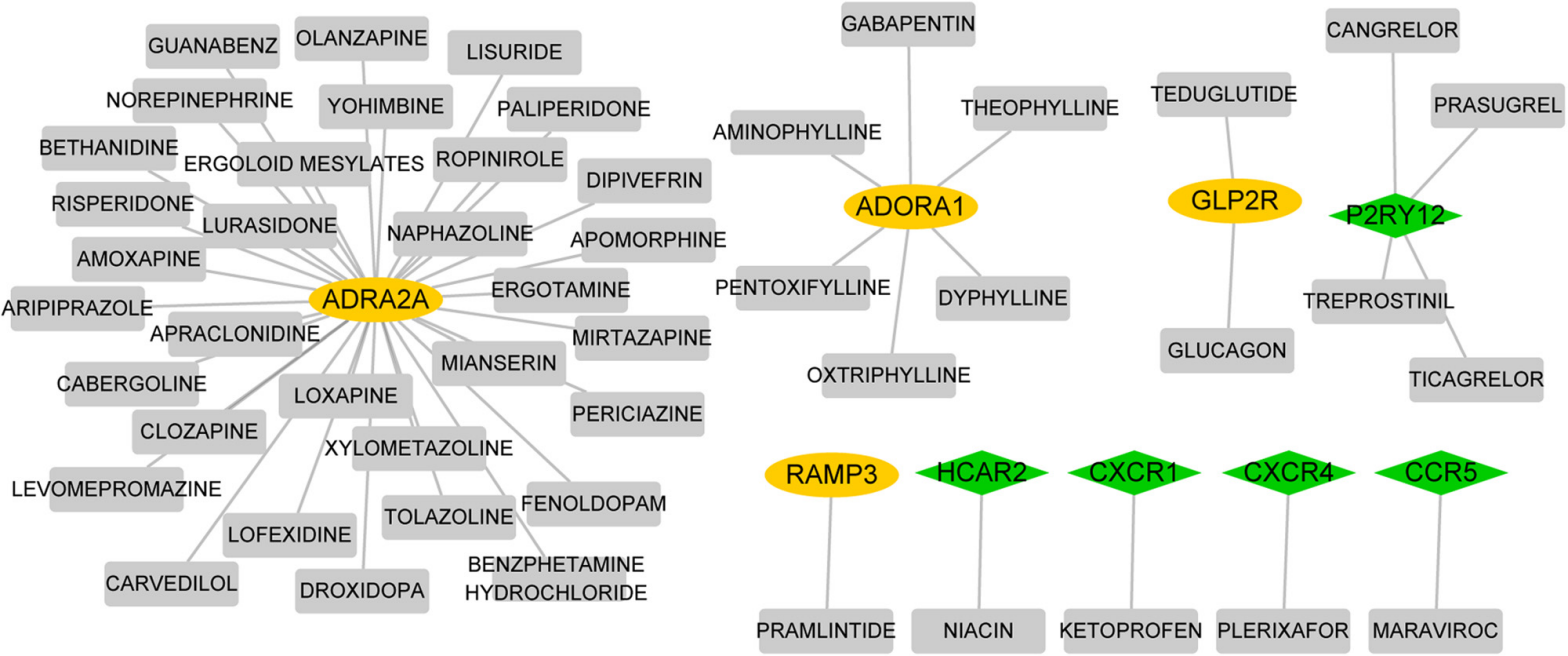
Network construction of drug–gene interaction. Yellow circle nodes are upregulated genes; gray square nodes are drug molecules. The yellow circle represents the upregulated gene, the green prismatic represents downregulated gene.

Based on the prediction analysis with DGIdb, 49 drug–gene interaction pairs were extracted, which contained four upregulated genes (*ADRA2A*, *ADORA1*, *GLP2R*, and *RAMP3*), five downregulated genes (*P2RY12*, *HCAR2*, *CXCR1*, *CXCR4*, and *CCR5*), and 49 kinds of drug molecules (Figure 6).

### 3.6 Association of genes with lung cancer prognosis

**Figure 7:**
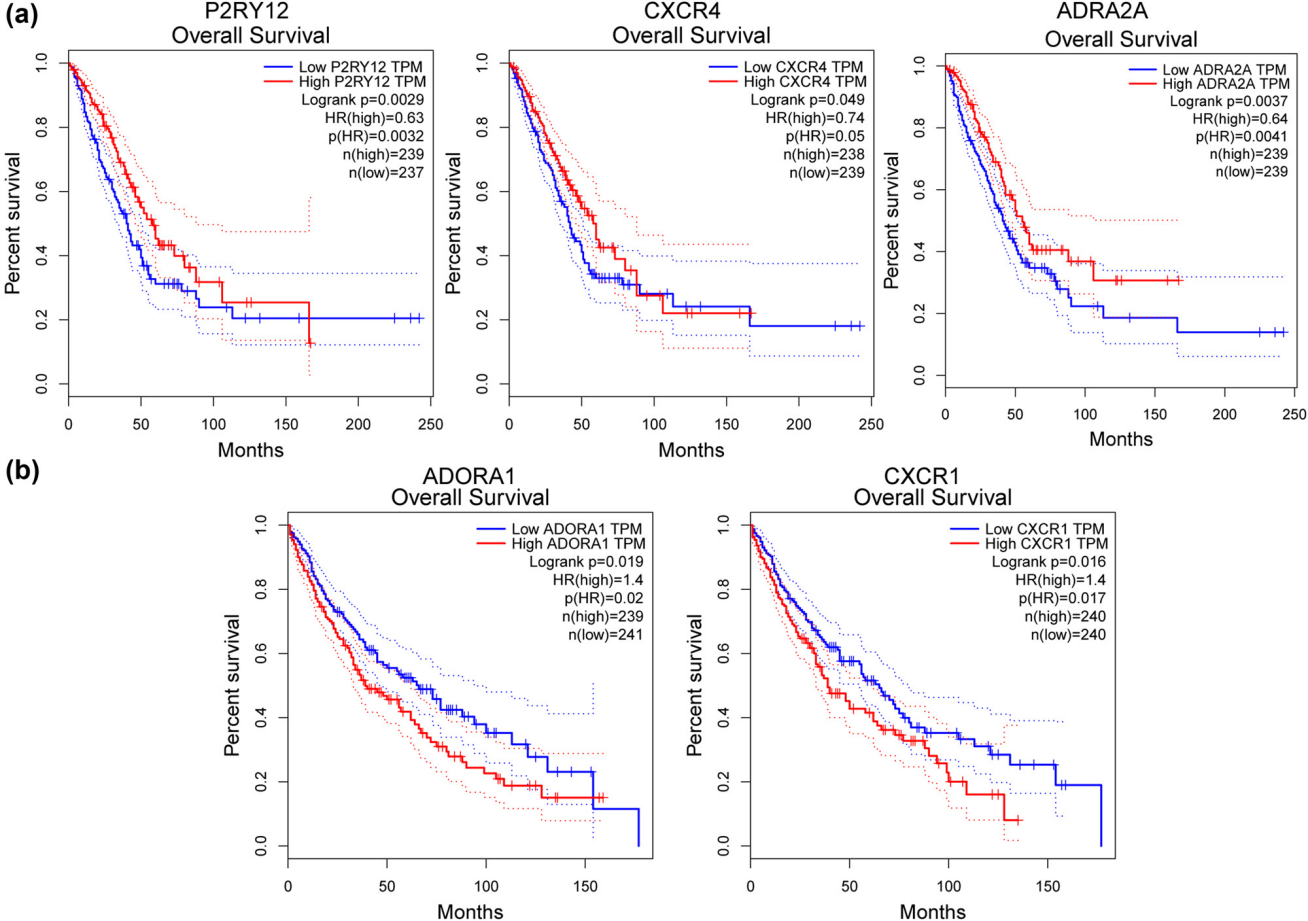
The lung cancer prognosis-related genes in ALI. The LUAD and LUSC data in the GEPIA online tool (http://gepia.cancer-pku.cn/) were employed for the survival analysis. (a) LUAD and (b) LUSC.

Using the LUAD and LUSC data in GEPIA online tool, the correlations of the nine DEGs in the drug–gene interaction network were analyzed. Results showed that the high expression levels of *P2RY12* (logrank *p* = 0.0029), *CXCR4* (logrank *p* = 0.049), and *ADRA2A* (logrank *p* = 0.0037) were significantly correlated with a good prognosis of patients with LUAD (Figure 7a), while the low expression levels of *ADORA1* (logrank *p* = 0.019) and *CXCR1* (logrank *p* = 0.016) were obviously related to higher survival ratios in LUSC patients (Figure 7b). No other correlation was identified.

## 4 Discussion

Extensive studies have concentrated on illuminating pathogenesis of ALI via bioinformatics analyses over the past few years [22,23,24]. In the present study, 421 DEGs between ALI lung specimens from lung-specific GGPPS1-knockout mice and wild-type mice treated with LPS for 12 h were identified. The upregulated DEGs mainly enriched in the peroxisome proliferator-activated receptor (PPAR) signaling pathway, drug metabolism-cytochrome P450, fatty acid metabolic process, and lipid catabolic process, while downregulated DEGs were prominently related to cytokine–cytokine receptor interaction, hematopoietic cell lineage, T cell activation, and regulation of cytokine production. *Cxcl9*, *Ccr5*, *Cxcr4*, and *Cxcl5* were key nodes in the PPI network. In addition, three miRNAs (miR505, miR23A, and miR23B) and three TFs (PU1, CEBPA, and CEBPB) were key molecules in the miRNA-TF-target network. Finally, several genes, such as *ADRA2A*, *P2RY12*, *ADORA1*, *CXCR1*, and *CXCR4*, were predicted as potential druggable genes for ALI with GGPPS1-knockout, and were associated with the prognosis of lung cancers.

Accumulating evidence has demonstrated that the main mechanisms of ALI are cyclic atelectasis and alveolar overdistention, which contribute to the activation of inflammatory cells and further aggravate ALI [25]. GGPPS1 is a catalase downstream of the mevalonate pathway, which is known for the synthesis of cholesterol and considered as a target to treat COVID-19 [10,11,26]. GGPPS1 is a key enzyme that has been reported to be highly expressed and involved in the pathogenesis of inflammatory diseases, including idiopathic pulmonary fibrosis [12], LPS-induced ALI [13], cigarette smoke-induced inflammation [27], and LUAD [28], through increasing the productions of inflammatory cytokines. The high expression level of GGPPS1 is considered to be responsible for the development of alveoli and airways in the fetal lung [29]. In this study, we found that *Cxcl9*, *Ccr5*, *Cxcr4*, and *Cxcl5* were identified as key genes for the function of GGPPS1-knockout in ALI. These genes participated in ALI by regulating inflammation and immune responses.

CXC chemokine ligands (including upregulated CXCL9 and CXCL5) and chemokine receptors (including downregulated CXCR1, CCR5, and CXCR4) are the members of chemokine family [30]. Research showed that the knockout of GGPPS1 attenuates lung inflammation and LPS-induced ALI [13,14]. The study by Xu et al. [13] showed that lung-specific GGPPS1-knockout decreased interleukin (IL)-1β level, cleaved caspase-3 expression, and apoptotic cell percentage in lung tissues in LPS-induced ALI mice. The interactions of chemokines and their receptors can participate in a variety of physiological functions, such as cell growth, development, differentiation, apoptosis, and distribution, and play important roles in a variety of pathological processes, including inflammation, pathogen infection, trauma repair, and tumor formation and metastasis [30,31]. Mevalonate promotes differentiation and proliferation of multiple types of cells, including colon cancer cells [26], cardiomyocytes [32], vascular smooth muscle cells [33], and regulatory T (Treg) cells [34]. The mevalonate pathway is essential for the growth and proliferation of cancer cells and energy homeostasis by controlling the uptake of glucose and amino acid [26,33], and the inhibition of mevalonate suppresses glucose and amino acid uptake in colon cancer cells [26]. The results showed that the downregulation of GGPPS1 suppressed mevalonate-mediated energy homeostasis and cell proliferation.

As reported, the *cxcl5* and *cxcl1* genes and inflammatory cytokines, including IL-6 and IL-1β, were significantly increased in ALI mice compared with controls [35]. Besides, excessive neutrophil responses result in life-threatening injury in the lung. Chemokines like CXCL5, CXCL9, CXCL10, and CXCL11 are upregulated following SARS-CoV-2 infection, ALI, or ARDS [36,37,38,39]. Elevated CXCL5 drives neutrophil recruitment and harms lung barrier function [38,39]. Berger et al. showed that reduced neutrophil numbers resulted in increased burden of *Streptococcus pneumoniae* infection in *Cxcl5* ^‒/‒^ mice and *Cxcl5* absence resulted in reduced alveolar neutrophil recruitment and decreased vascular leakage compared with wild-type mice [39]. In addition, CXCR4 overexpression in mesenchymal stem cells can improve the therapeutic effect for ALI [40]. These data showed that the immune responses were crucial for the development and pathogenesis of lung diseases including ALI. Also, targeting chemokines and chemokine receptors, including CXCL9, CXCL5, CXCR1, CCR5, and CXCR4, may possibly provide a therapeutic perspective in ALI.

The α-2A adrenergic receptor (*ADRA2A*) gene mainly functions in the central nervous system through the regulation of neurotransmitter released by adrenergic neurons [41,42]. It has been reported to be associated with attention-deficit hyperactivity disorder [43]. The adenosine A1 receptor subtype gene (*ADORA1*) plays a protective role against hypoxia damage in cells via regulating the level of adenosine, which controls both the inflammation and neurodegeneration [44,45]. It also involves in the central nervous system disease including the pathogenesis of parkinsonism and cognitive dysfunction [46]. A recent study by Valasarajan et al. [47] showed that the dysregulation of *ADORA1* was involved in pulmonary hypertension. Also, the positive modulation of adenosine and adenosine kinase on inflammation has been confirmed in endothelial [48]. However, there is less information on the association between these genes with inflammation responses in lung diseases. In our study, the upregulation of *ADORA1* and *ADRA2A* in ALI lung tissues were confirmed. They also correlated with the prognosis of LUSC and LUAD, respectively. These results highlighted the potential involvement or contribution of them to ALI.

Based on the miRNA-TF-target network, TFs, such as PU1, CEBPA, and CEBPB, were significant regulated molecules for the role of GGPPS1-knockout in ALI in the current study. PU1 as a hematopoietic lineage-specifying TF has been reported to play important roles in regulating gene expression of various immune cells, including B-cell, dendritic-cell, granulocyte, and macrophage [49]. Several research studies have suggested that PU1 can promote inflammatory response in many inflammatory diseases, including allergic inflammation [50], asthmatic airway inflammation [51], and pulmonary inflammation response to LPS [52]. In addition, CEBPA and CEBPB initially have been revealed to exert functions in adipogenesis and hematopoiesis. Recent studies have shown that methylation of CEBPA promoter exerts positive regulating role in lung inflammation [53]. Interestingly, both CEBPA and CEBPB can play a role in the collaboration of PU1, and the binding of PU1 to CEBPA/CEBPB may be involved in the inflammatory response by activating macrophages [54,55].

Furthermore, our data analysis identified that three miRNAs (miR505, miR23A, and miR23B) are important molecules of GGPPS1 knockout-induced ALI. Previous study has shown that miR-505-3p may participate in chronic inflammation through upregulating chemokine receptors, such as CCR3, CCR4, and CXCR1 [56]. Additionally, miR23A and miR23B also proved to be able to modulate the inflammatory response [57,58,59,60]. The miR-23b-3p expression is downregulated and cytokines of IL-1β, IL-6, IL-4, and IL-8 are upregulated in LPS-induced ALI mice [57]. The overexpression of miR-23b-3p counteracted LPS-induced effects in ALI mice, which were intensified by miR-23b-3p downregulation [57]. A study by Chen et al. [58] showed that LPS induced miR-23a-5p expression in the lungs from ALI mice, and enhanced inflammation, oxidative stress, lung tissue injury, and pulmonary dysfunction in LPS-induced ALI mice by targeting heat shock protein 20 -apoptosis signal-regulating kinase 1 [58]. They found that miR-23a-5p agomir aggravated pulmonary inflammation, increased pulmonary oxidative damage, and activated nucleotide-binding domain-like receptor protein 3 inflammasome in ALI mice. In contrast, miR-23a-5p antagomir blocked LPS-induced inflammation and oxidative stress in macrophages [58]. Thus, these data indicated that the three miRNAs (miR505, miR23A, and miR23B) play crucial roles in ALI by regulating inflammatory response.

## 5 Conclusion

In conclusion, 421 DEGs between ALI tissue specimens from lung-specific GGPPS1-knockout and wild-type mice were identified. Among these DEGs, the genes such as *ADRA2A*, *P2RY12*, *ADORA1*, *CXCR1*, and *CXCR4* might be novel markers and potential druggable genes in ALI by regulating inflammatory response, which might be regulated by miRNAs (miR505, miR23A, and miR23B) and TFs (PU1, CEBPA, and CEBPB).

## References

[1] Butt Y, Kurdowska A, Allen TC. Acute lung injury: a clinical and molecular review. Arch Pathol Lab Med. 2016;140(4):345–50. 10.5858/arpa.2015-0519-RA 27028393

[2] Johnson ER, Matthay MA. Acute lung injury: epidemiology, pathogenesis, and treatment. J Aerosol Med Pulm D. 2010;23(4):243–52. 10.1089/jamp.2009.0775 PMC3133560 20073554

[3] Cai A, McClafferty B, Benson J, Ramgobin D, Kalayanamitra R, Shahid Z, et al. COVID-19: Catastrophic cause of acute lung injury. South Dak Med. 2020;73:6. 32580257

[4] Li L, Huang Q, Wang DC, Ingbar DH, Wang X. Acute lung injury in patients with COVID-19 infection. Clin Transl Med. 2020;10(1):20–7. 10.1002/ctm2.16 PMC7240840 32508022

[5] Standiford TJ, Ward PA. Therapeutic targeting of acute lung injury and acute respiratory distress syndrome. Transl Res. 2016;167(1):183–91. 10.1016/j.trsl.2015.04.015 PMC4635065 26003524

[6] Fanelli V, Ranieri VM. Mechanisms and clinical consequences of acute lung injury. Ann Am Thorac Soc. 2015;12(Supplement 1):S3–8. 10.1513/AnnalsATS.201407-340MG 25830831

[7] Beermann J, Piccoli M-T, Viereck J, Thum T. Non-coding RNAs in development and disease: background, mechanisms, and therapeutic approaches. Physiol Rev. 2016;96(4):1297–325. 10.1152/physrev.00041.2015 27535639

[8] Piletič K, Kunej T. MicroRNA epigenetic signatures in human disease. Arch Toxicol. 2016;90(10):2405–19. 10.1007/s00204-016-1815-7 27557899

[9] Rajasekaran S, Pattarayan D, Rajaguru P, Sudhakar Gandhi P, Thimmulappa RK. MicroRNA regulation of acute lung injury and acute respiratory distress syndrome. J Cell Physiol. 2016;231(10):2097–106. 10.1002/jcp.25316 26790856

[10] Singla S, Jacobson JR. Statins as a novel therapeutic strategy in acute lung injury. Pul Circ. 2012;2(4):397–406. 10.4103/2045-8932.105028 PMC3555410 23372924

[11] Fajgenbaum DC, Rader DJ. Teaching old drugs new tricks: statins for COVID-19? Cell Metab. 2020;32(2):145–7. 10.1016/j.cmet.2020.07.006 PMC7402379 32755604

[12] Chen M, Wan B, Zhu S, Zhang F, Jin J, Li X, et al. Geranylgeranyl diphosphate synthase deficiency aggravates lung fibrosis in mice by modulating TGF-β1/BMP-4 signaling. Biol Chem. 2019;400(12):1617–27. 10.1515/hsz-2019-0168 31120854

[13] Xu W-J, Wang X-X, Jin J-J, Zou Q, Wu L, Lv T-F, et al. Inhibition of GGPPS1 attenuated LPS-induced acute lung injury and was associated with NLRP3 inflammasome suppression. Am J Physiol-Lung C. 2019;316(3):L567–77. 10.1152/ajplung.00190.2018 30652497

[14] Wan B, Xu W-J, Chen M-Z, Sun S-S, Jin Y-L, Lv J-J, et al. Geranylgeranyl diphosphate synthase 1 knockout ameliorates ventilator-induced lung injury via regulation of TLR2/4-AP-1 signaling. Free Radic Bio Med. 2019;147:159–66. 10.1016/j.freeradbiomed.2019.12.024 31874250

[15] Smyth GK. Limma: linear models for microarray data. In: Bioinformatics and computational biology solutions using R and Bioconductor (pp. 397–420). New York, NY: Springer; 2005.

[16] Yu G, Wang L-G, Han Y, He Q-Y. clusterProfiler: an R package for comparing biological themes among gene clusters. OMICS. 2012;16(5):284–7. 10.1089/omi.2011.0118 PMC3339379 22455463

[17] Szklarczyk D, Franceschini A, Wyder S, Forslund K, Heller D, Huerta-Cepas J, et al. STRING v10: protein–protein interaction networks, integrated over the tree of life. Nucleic Acids Res. 2014;43(D1):D447–D52. 10.1093/nar/gku1003 PMC4383874 25352553

[18] Tang Y, Li M, Wang J, Pan Y, Wu F-X. CytoNCA: a cytoscape plugin for centrality analysis and evaluation of protein interaction networks. Biosystems. 2015;127:67–72. 10.1016/j.biosystems.2014.11.005 25451770

[19] Bandettini WP, Kellman P, Mancini C, Booker OJ, Vasu S, Leung SW, et al. MultiContrast Delayed Enhancement (MCODE) improves detection of subendocardial myocardial infarction by late gadolinium enhancement cardiovascular magnetic resonance: a clinical validation study. J Cardiovascular Magnetic Reson. 2012;14(1):83. 10.1186/1532-429X-14-83 PMC3552709 23199362

[20] Zhang B, Kirov S, Snoddy J. WebGestalt: an integrated system for exploring gene sets in various biological contexts. Nucleic Acids Res. 2005;33(suppl_2):W741–8. 10.1093/nar/gki475 PMC1160236 15980575

[21] Wagner AH, Coffman AC, Ainscough BJ, Spies NC, Skidmore ZL, Campbell KM, et al. DGIdb 2.0: mining clinically relevant drug–gene interactions. Nucleic Acids Res. 2015;44(D1):D1036–44. 10.1093/nar/gkv1165 PMC4702839 26531824

[22] Wang J, Shen Y-C, Chen Z-N, Yuan Z-C, Wang H, Li D-J, et al. Microarray profiling of lung long non-coding RNAs and mRNAs in lipopolysaccharide-induced acute lung injury mouse model. Biosci Rep. 2019;39(4):BSR20181634. 10.1042/BSR20181634 PMC6488857 30979832

[23] dos Santos CC, Okutani D, Hu P, Han B, Crimi E, He X, et al. Differential gene profiling in acute lung injury identifies injury-specific gene expression. Crit Care Med. 2008;36(3):855–65. 10.1097/CCM.0B013E3181659333 18431273

[24] Fang X, Bai C, Wang X. Bioinformatics insights into acute lung injury/acute respiratory distress syndrome. Clin Transl Med. 2012;1(1):9. 10.1186/2001-1326-1-9. PMC3560991 23369517

[25] Luh S-P, Chiang C-H. Acute lung injury/acute respiratory distress syndrome (ALI/ARDS): the mechanism, present strategies and future perspectives of therapies. J Zhejiang Univ Sci B. 2007;8(1):60–9. 10.1631/jzus.2007.B0060 PMC1764923 17173364

[26] Gong L, Xiao Y, Xia F, Wu P, Zhao T, Xie S, et al. The mevalonate coordinates energy input and cell proliferation. Cell Death Dis. 2019;10(4):1–14. 10.1038/s41419-019-1544-y PMC6459916 30975976

[27] Shen N, Gong T, Wang J-D, Meng F-L, Qiao L, Yang R-L, et al. Cigarette smoke–induced pulmonary inflammatory responses are mediated by EGR-1/GGPPS/MAPK signaling. Am J Pathol. 2011;178(1):110–8. 10.1016/j.ajpath.2010.11.016 PMC3069843 21224049

[28] Wang X, Xu W, Zhan P, Xu T, Jin J, Miu Y, et al. Overexpression of geranylgeranyl diphosphate synthase contributes to tumour metastasis and correlates with poor prognosis of lung adenocarcinoma. J Cell Mol Med. 2018;22(4):2177–89. 10.1111/jcmm.13493 PMC5867137 29377583

[29] Jia W-J, Jiang S, Tang Q-L, Shen D, Xue B, Ning W, et al. Geranylgeranyl diphosphate synthase modulates fetal lung branching morphogenesis possibly through controlling K-Ras prenylation. Am J Pathol. 2016;186(6):1454–65. 10.1016/j.ajpath.2016.01.021 27106761

[30] Yoshie O, Matsushima K. Chemokines and Chemotaxis. In: Cavaillon J-M., Singer M., editors. Inflammation; 2017. p. 619–50. 10.1002/9783527692156.ch25.

[31] Legler DF, Thelen M. Chemokines: chemistry, biochemistry and biological function. CHIMIA. 2016;70(12):856–9. 10.2533/chimia.2016.856 28661356

[32] Edwards W, Greco TM, Miner GE, Barker NK, Herring L, Cohen S, et al. Quantitative proteomic profiling of murine embryonic heart development reveals a role for the mevalonate pathway in cardiomyocyte proliferation. bioRxiv. 2022. 10.1101/2022.02.21.481309.

[33] Ye D, Lou GH, Li AC, Dong FQ, Chen GP, Xu WW, et al. MicroRNA-125a-mediated regulation of the mevalonate signaling pathway contributes to high glucose-induced proliferation and migration of vascular smooth muscle cells. Mol Med Rep. 2020;22(1):165–74. 10.3892/mmr.2020.11077 PMC7248521 32319638

[34] Acharya S, Timilshina M, Chang J-H. Mevalonate promotes differentiation of regulatory T cells. J Mol Med. 2019;97(7):927–36. 10.1007/s00109-019-01784-y 31020340

[35] Störmann P, Becker N, Künnemeyer L, Wutzler S, Vollrath JT, Lustenberger T, et al. Contributing factors in the development of acute lung injury in a murine double hit model. Eur J Trauma Emerg S. 2020;46(1):21–30. 10.1007/s00068-019-01121-5 30937460

[36] Callahan V, Hawks S, Crawford MA, Lehman CW, Morrison HA, Ivester HM, et al. The pro-inflammatory chemokines CXCL9, CXCL10 and CXCL11 are upregulated following SARS-CoV-2 infection in an AKT-dependent manner. Viruses. 2021;13(6):1062. 10.3390/v13061062 PMC8226769 34205098

[37] Wang C-y, Shang M, Zhou C-l, Feng L-z, Zhou Q-s, Hu K. Mechanism of Cxc chemokine ligand 5 (CXCL5)/Cxc chemokine receptor 2 (CXCR2) bio-axis in mice with acute respiratory distress syndrome. Med Sci Monit. 2019;25:5299–305. 10.12659/MSM.915835 PMC6659456 31311916

[38] Nouailles G, Berger S, Wienhold S, Goekeri C, Behrendt U, Muller-Redetzky H, et al. CXCL5 drives neutrophil recruitment and development of lung barrier failure in acute lung injury. B31 Acute lung injury and ARDS: translational and mechanistic studies. Am Thorac Soc. 2018;A2962-A.

[39] Berger S, Wienhold S-M, Goekeri C, Behrendt U, Müller-Redetzky H, Dietert K, et al. CXCL5-dependent neutrophil recruitment harms lung barrier function in acute lung injury. Eur Resp Soc. 2018;52:PA4295. 10.1183/13993003.congress-2018.PA4295.

[40] Yang J-X, Zhang N, Wang H-W, Gao P, Yang Q-P, Wen Q-P. CXCR4 receptor overexpression in mesenchymal stem cells facilitates treatment of acute lung injury in rats. J BiolChem. 2015;290(4):1994–2006. 10.1074/jbc.M114.605063 PMC4303655 25492872

[41] Huang HC, Wu LS, Yu SC, Wu BJ, Lua AC, Lee SM, et al. The alpha-2A adrenergic receptor gene-1291C/G single nucleotide polymorphism is associated with the efficacy of methylphenidate in treating Taiwanese children and adolescents with attention-deficit hyperactivity disorder. Psychiat Invest. 2018;15(3):306–12. 10.30773/pi.2017.07.24 PMC5900374 29486545

[42] Fetterly TL, Basu A, Nabit BP, Awad E, Williford KM, Centanni SW, et al. α2A-adrenergic receptor activation decreases parabrachial nucleus excitatory drive onto BNST crf neurons and reduces their activity in vivo. J Neurosci. 2019;39(3):472–84. 10.1523/JNEUROSCI.1035-18.2018 PMC6335747 30478032

[43] Roman T, Schmitz M, Polanczyk GV, Eizirik M, Rohde LA, Hutz MH. Is the alpha-2A adrenergic receptor gene (ADRA2A) associated with attention-deficit/hyperactivity disorder. Am J Med Genet B. 2003;120B(1):116–20. 10.1002/ajmg.b.20018 12815749

[44] Meng F, Guo Z, Hu Y, Mai W, Zhang Z, Zhang B, et al. CD73-derived adenosine controls inflammation and neurodegeneration by modulating dopamine signalling. Brain. 2019;142(3):700–18. 10.1093/brain/awy351 30689733

[45] Mei HF, Poonit N, Zhang YC, Ye CY, Cai XH. Activating adenosine A1 receptor accelerates PC12 cell injury via ADORA1/PKC/KATP pathway after intermittent hypoxia exposure. Mol Cell Biochem. 2018;446(2):1–10. 10.1007/s11010-018-3283-2 29380238

[46] Jaberi E, Rohani M, Shahidi GA, Nafissi S, Arefian E, Soleimani M, et al. Mutation in ADORA1 identified as likely cause of early-onset parkinsonism and cognitive dysfunction. Mov Disord. 2016;31(7):1004–11. 10.1002/mds.26627 27134041

[47] Valasarajan C, Laria JCP, Laria NCP, Wietelmann A, Grimminger F, Seeger W, et al. Targeting ADORA1/PDE10A signalosome regulated cAMP microenvironment as a novel therapeutic approach for treating pulmonary hypertension. Am J Resp Crit Care. 2019;199:A4392.

[48] Xu Y, Wang Y. Regulation of endothelial intracellular adenosine via adenosine kinase epigenetically modulates vascular inflammation. Nat Commun. 2017;8(1):943. 10.1038/s41467-017-00986-7. PMC5643397 29038540

[49] Carotta S, Wu L, Nutt SL. Surprising new roles for PU. 1 In the adaptive immune response. Immunol Rev. 2010;238(1):63–75. 10.1111/j.1600-065X.2010.00955.x 20969585

[50] Chang H-C, Sehra S, Goswami R, Yao W, Yu Q, Stritesky GL, et al. The transcription factor PU. 1 is required for the development of IL-9-producing T cells and allergic inflammation. Nat Immunol. 2010;11(6):527–34. 10.1038/ni.1867 PMC3136246 20431622

[51] Qian F, Deng J, Lee YG, Zhu J, Karpurapu M, Chung S, et al. The transcription factor PU. 1 promotes alternative macrophage polarization and asthmatic airway inflammation. J Mol Cell Biol. 2015;7(6):557–67. 10.1093/jmcb/mjv042 PMC4664118 26101328

[52] Berclaz P-Y, Carey B, Fillipi M-D, Wernke-Dollries K, Geraci N, Cush S, et al. GM-CSF regulates a PU. 1-Dependent transcriptional program determining the pulmonary response to LPS. Am J Resp Cell Mol. 2007;36(1):114–21. 10.1165/rcmb.2006-0174OC PMC1899305 16917076

[53] Wang N, Li Q, Liu H, Lin L, Han W, Hao W. Role of C/EBPα hypermethylation in diesel engine exhaust exposure-induced lung inflammation. Ecotox Env Safe. 2019;183:109500. 10.1016/j.ecoenv.2019.109500 31450033

[54] Feng R, Desbordes SC, Xie H, Tillo ES, Pixley F, Stanley ER, et al. PU. 1 and C/EBPα/β convert fibroblasts into macrophage-like cells. PNAS. 2008;105(16):6057–62. 10.1073/pnas.0711961105 PMC2327209 18424555

[55] Ponomarev ED, Veremeyko T, Barteneva N, Krichevsky AM, Weiner HL. MicroRNA-124 promotes microglia quiescence and suppresses EAE by deactivating macrophages via the C/EBP-α–PU. 1 pathway. Nat Med. 2011;17(1):64–70. 10.1038/nm.2266 PMC3044940 21131957

[56] Escate R, Mata P, Cepeda JM, Padró T, Badimon L. miR-505-3p controls chemokine receptor up-regulation in macrophages: role in familial hypercholesterolemia. FASEB J. 2017;32(2):601–12. 10.1096/fj.201700476R. 29457550

[57] Zhang P, Liu L, Yao L, Song X. Improved differentiation ability and therapeutic effect of miR-23a-3p expressing bone marrow-derived mesenchymal stem cells in mice model with acute lung injury. Int J Stem Cell. 2021;14(2):229–39. 10.15283/ijsc20136. PMC8138660 33632989

[58] Chen Y-F, Hu F, Wang X-G, Tang Z, Tang H-X, Xu M. MicroRNA-23a-5p Is Involved in the regulation of lipopolysaccharide-induced acute lung injury by targeting HSP20/ASK1. Oxid Med Cell Longev. 2021;2021:9942557. 10.1155/2021/9942557 PMC8376430 34422215

[59] Si X, Cao D, Chen J, Nie Y, Jiang Z, Chen MY, et al. miR-23a downregulation modulates the inflammatory response by targeting ATG12-mediated autophagy. Mol Med Rep. 2018;18(2):1524–30. 10.3892/mmr.2018.9081 PMC6072189 29845275

[60] Zhang W, Lu F, Xie Y, Lin Y, Zhao T, Tao S, et al. miR-23b negatively regulates sepsis-induced inflammatory responses by targeting ADAM10 in human THP-1 monocytes. Mediat Inflamm. 2019;2019:5306541. 10.1155/2019/5306541 PMC6875296 31780861

